# The Origin of the Frequency-Dependence of OH Stretch Vibrational Lifetimes in Liquid Water

**DOI:** 10.1021/acs.jpclett.6c00970

**Published:** 2026-07-27

**Authors:** Marcin Pastorczak, Czesław Radzewicz, Aritri Biswas, Alexei A. Kananenka

**Affiliations:** ◧ Institute of Physical Chemistry, 119463 Polish Academy of Sciences, Kasprzaka 44/52, 01-224 Warsaw, Poland; ‡ Institute of Experimental Physics, Faculty of Physics, 226285 University of Warsaw, Pasteura 5, 02-093 Warsaw, Poland; § Department of Physics and Astronomy, 5972 University of Delaware, Newark, Delaware 19716, United States

## Abstract

Polarization-resolved infrared pump-stimulated Raman probe spectroscopy was used to measure the lifetimes of OH stretching vibrations in liquid water, giving rise to polarized and depolarized Raman signals. The lifetime of the OH stretch for the polarized Raman was determined to be 200 fs, in excellent agreement with previous measurements. The lifetime of OH stretches contributing to the depolarized Raman signal was determined here for the first time and was found to be at least two times longer (≥400 fs). With the support of mixed quantum-classical line shape calculations, the experimental lifetimes were rationalized in terms of the extent of the coupling between the OH stretching modes as well as their coupling to the overtone of water bending vibrations. OH stretches that participate in an extensive network of coupled vibrations contribute to the polarized Raman spectrum, while mainly uncoupled OH modes give rise to the depolarized Raman signals.

## Graphical abstract

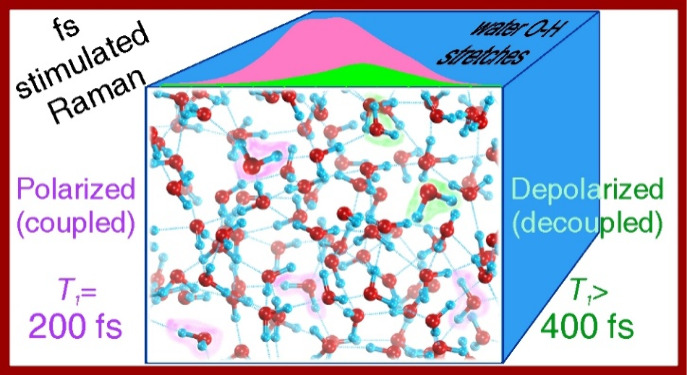

Water is the most important substance on Earth and the major component of human bodies. The desire to understand the vibrational energy relaxation (VER) of neat water has been motivating numerous ultrafast spectroscopic studies for decades. The rate of vibrational relaxation of water impacts the catalysis in the aqueous environment^1,2^ and fluorophores’ quantum yields.^3^ VER times of HDO/H_2_O or HDO/D_2_O systems are used to study the hydration of proteins,^4^ other biomolecules,^5^ polymers,^6^ and electrolytes.^7^ Furthermore, the supramolecular structure of liquid water has been debated for over a century.^8^ The OH stretching band of liquid water spans ca. 3000–3900 cm^–1^ range, featuring structural heterogeneity due to various hydrogen-bonding environments, combination bands, and stretch–bend Fermi resonance. Therefore, the question of whether such a complex structure causes heterogeneity in OH-stretch vibrational relaxation times became a critical issue in understanding vibrational spectroscopy and the structure of water. IR pump-spontaneous Raman probe experiments^9,10^ proposed the two vibrational “substructures” but were later concluded to result from an insufficient (around 1 ps) temporal resolution of the method.^11^ Thus, for years, it had been believed that water OH stretch vibrations have homogeneous lifetimes.^12–16^ This view changed when van der Post and co-workers showed the strong frequency-dependence of the OH stretch lifetimes in both bulk water and at the air/water interface.^17^ Recently, Sung et al. reported time-resolved, heterodyne-detected vibrational sum-frequency generation (TR-HD-SFG) measurements that contradict the frequency dependence of VER at the interface.^18^ 2D IR studies by de Marco et al. pointed at only minor frequency dependence in the bulk water (220 fs at the red vs 310 fs at the blue side of the OH stretching band).^19^

In this work, we address this decades-long debate using the recently developed femtosecond IR pump–stimulated Raman probe spectroscopy (fs-IR-SRS).^20,21^ Polarization-dependence of the response, which effectively serves as a selection rule, is a salient advantage of polarization-resolved Raman spectroscopy, making fs-IR-SRS a well-suited probe of the frequency-dependence of the OH stretching VER in various environments. The polarized Raman spectrum of water is associated with the isotropic part of the polarizability tensor and its highly polarized low-frequency component (around 3250 cm^–1^) is dominated by the “collective” (coupled) OH stretch vibrations.^22,23^ The blue-shifted “depolarized” component of the Raman spectrum of water (see Figure 1) is associated with the anisotropic part of the polarizability tensor. Traditionally, it was assigned to the antisymmetric (*ν*_3_) OH stretches, based on the analogy to the vibrations of gas-phase water.^24,25^ However, recent theoretical^22,26,27^ and experimental^14,28^ works show evidence of a significant delocalization of the OH stretches in the liquid and solid water, making the distinction of OH stretching modes into symmetric (*ν*_1_) and antisymmetric (*ν*_3_) inadequate in the condensed phase. Therefore, we assign the depolarized Raman spectrum of water to the fraction of OH stretches that are decoupled from the rest of OH stretches. Such a picture can be further supported by a simple experiment in which we compare the polarized and depolarized Raman spectra of the decoupled OH oscillators (3 mol % H_2_O in D_2_O) with the depolarized Raman spectrum of neat H_2_O (Figure 1A). After intensity normalization, the decoupled polarized and depolarized spectra have nearly identical line shapes and are both very similar to the depolarized spectrum of neat liquid H_2_O. All three spectra are also blue-shifted to around 3500 cm^–1^. It thus shows that the orthogonal (*sp*) Raman probe polarization is an efficient way to single out the fraction of water OH oscillators that are decoupled from others due to their local environment (weakly hydrogen-bonded or “free” OHs).

**Figure 1:**
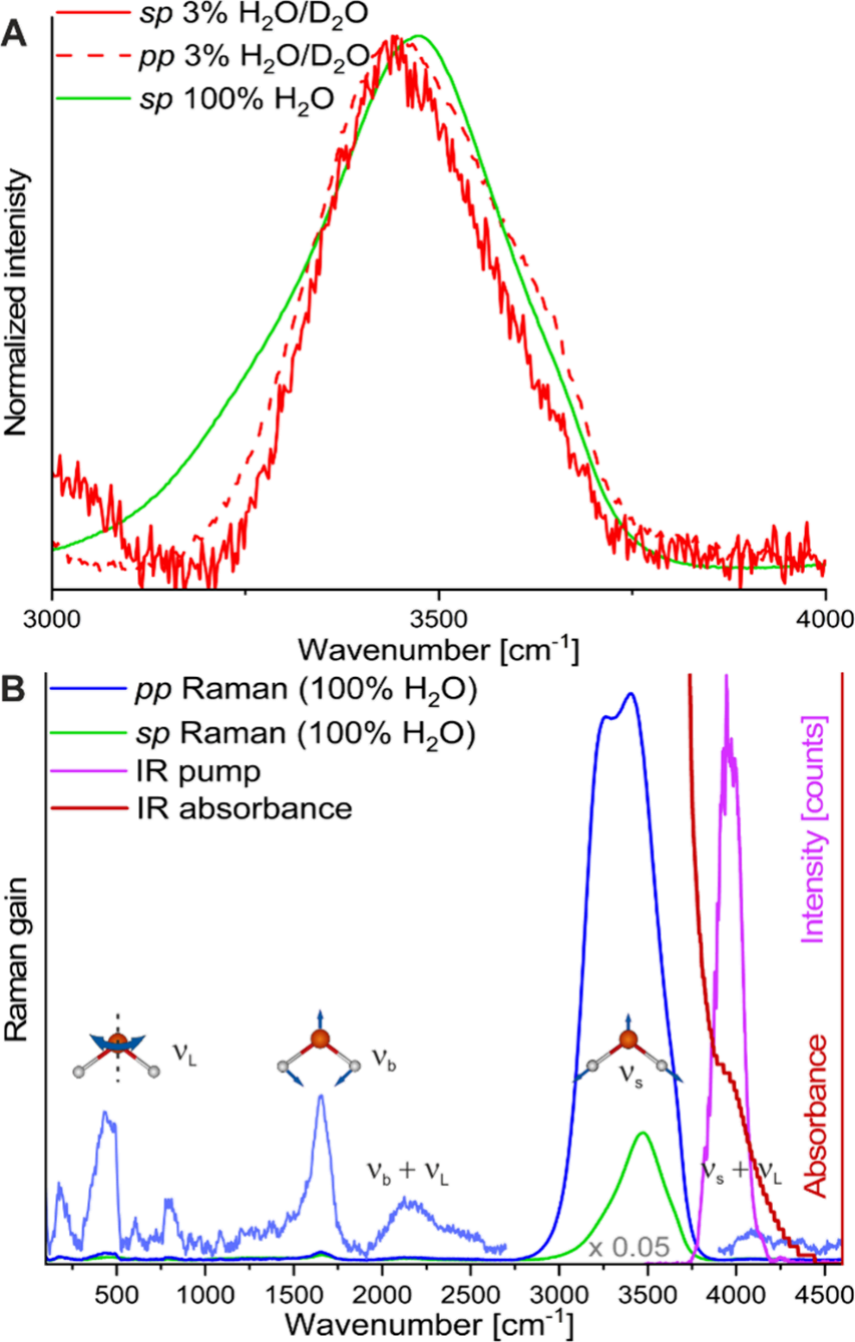
(A) Comparison of experimental *sp*/*pp* polarized Raman spectra of isotoptically diluted water (3 mol % H_2_O in D_2_O) with *sp* polarized Raman of neat H_2_O in the OH stretch spectral range. (B) Experimental pump (magenta), IR absorbance (dark red) and *pp* (blue)/*sp* (green) stimulated Raman spectra of water with the assignment of the selected vibrational modes.

In fs-IR-SRS, a fraction of molecules is promoted into a vibrationally excited state by a strong resonant IR pulse, and the transient state of a system is subsequently probed with femtosecond stimulated Raman. The resulting time-resolved spectra carry all the advantages of FSRS, i.e. around 10^5^ times stronger signals compared to spontaneous Raman, subpicosecond temporal (∼90 fs) and good spectral (5–10 cm^–1^) resolutions together with a multiplex advantage (approximately 100–4000 cm^–1^ spectral window in a single shot).^20^ Similarly to pump–probe IR, the transient Raman spectrum is the difference between the spectra obtained after and before the vibrational excitation. We also observe the depopulation of the vibrational ground state as a spectral “bleaching” or increased Raman scattering, by monitoring the vibrational 1→2 transition, which has a lower frequency than 0→1 transition due to vibrational anharmonicity (see Scheme S1 in Supporting Information, SI).

Here, we apply fs-IR-SRS to investigate the VER of the OH stretch vibrations in neat liquid H_2_O. We excited liquid water at around 2530 nm, corresponding to the weak IR-active combinational mode at 3950 cm^–1^ comprised of OH stretch and a libration.^29^ Subsequently, we observed transient stimulated Raman spectra for both polarized and depolarized configurations of the Raman pump (515 nm) and the probe (supercontinuum).

Liquid water exhibits enormous infrared absorption in the range of the OH stretching vibrations, with an absorption coefficient close to 12000 cm^–1^, meaning that infrared pump–probe experiments are typically performed on 1–2 μm thick water films. For thicker samples, the IR probe would be barely transmitted, and molecules would be excited only in the sample’s top layer. FSRS, despite its superior efficiency vs spontaneous Raman, requires a much thicker sample to produce a high-quality transient spectrum. Henceforth, we excited approximately 50 μm thick water sample flowing as a liquid jet with an IR pulse centered at 2530 nm, where the optical density (OD) of such a water layer is around 0.5. The corresponding IR absorption peak at 3950 cm^–1^ is assigned to water combinational band OH stretch + libration.^29^ Therefore, the energy absorption at this frequency should lead to nearly instantaneous excitation of both OH stretches (*ν*_s_) and water librations (*ν*_L_). As shown in Figure 1B, and in our previous study,^30^ this IR transition is either Raman inactive or the corresponding Raman peak is shifted to around 4050 cm^–1^. Hence, we do not expect any transient Raman signal at the pump wavenumber. Since we are only interested in studying the OH stretch VER, we neglect the kinetics of the energy relaxation from the 3950 cm^–1^ mode to the fundamental OH stretch. Following the excitation, we probed the stimulated Raman spectrum of the system for *pp* (parallel, “polarized” Raman spectrum) and *sp* (perpendicular, “depolarized” Raman spectrum) polarization configurations between a picosecond 515 nm Raman pump and fs Raman probe (supercontinuum). The parallel and perpendicular polarizations, with respect to the plane of incidence, are labeled “*p*” and “*s*”, respectively. The IR pump pulse polarization was kept *p*. Thus, overall, there were two polarization configurations *ppp* (for IR pump, Raman pump, Raman probe, respectively) and *psp*.

The transient *pp* Raman spectra in the range of the OH stretching vibrations for selected time delays are shown in Figure 2A. The spectra at short time delays are dominated by the signatures of the excited state (ES). They correspond well to typical water transient spectra from pump–probe IR experiments in this spectral range. At around 3600 cm^–1^, we observe a broad bleaching signal related to the depopulation of the vibrational ground state (GSB), and the increased Raman signal at around 3050 cm^–1^ is associated with the anharmonically shifted 1→2 OH stretch transition. At longer time delays (>0.4 ps), the shape of the spectra changes. We observe bleaching of the signal at shorter, around 3200 cm^–1^, and an increased signal at longer, around 3600 cm^–1^ wavenumbers. These spectral signatures are typically assigned to the thermalization of the sample associated with the relaxation of the vibrational energy, which leads to weakening of water hydrogen bonds and a shift of the OH stretching band to the blue. Usually, such a spectral component is related to the “heated ground state” (HGS) of water.^19,31^ Indeed, the transient *pp* Raman spectrum at long time delay (135 ps) corresponds well to the difference Raman spectrum (spectrum at 293 K subtracted from the spectrum at 301 K) obtained after heating the water sample by 8 K (Figure S1c), which agrees with the increase in the temperature of the sample produced by the energy of IR pulse used in the experiment (see SI file).

**Figure 2:**
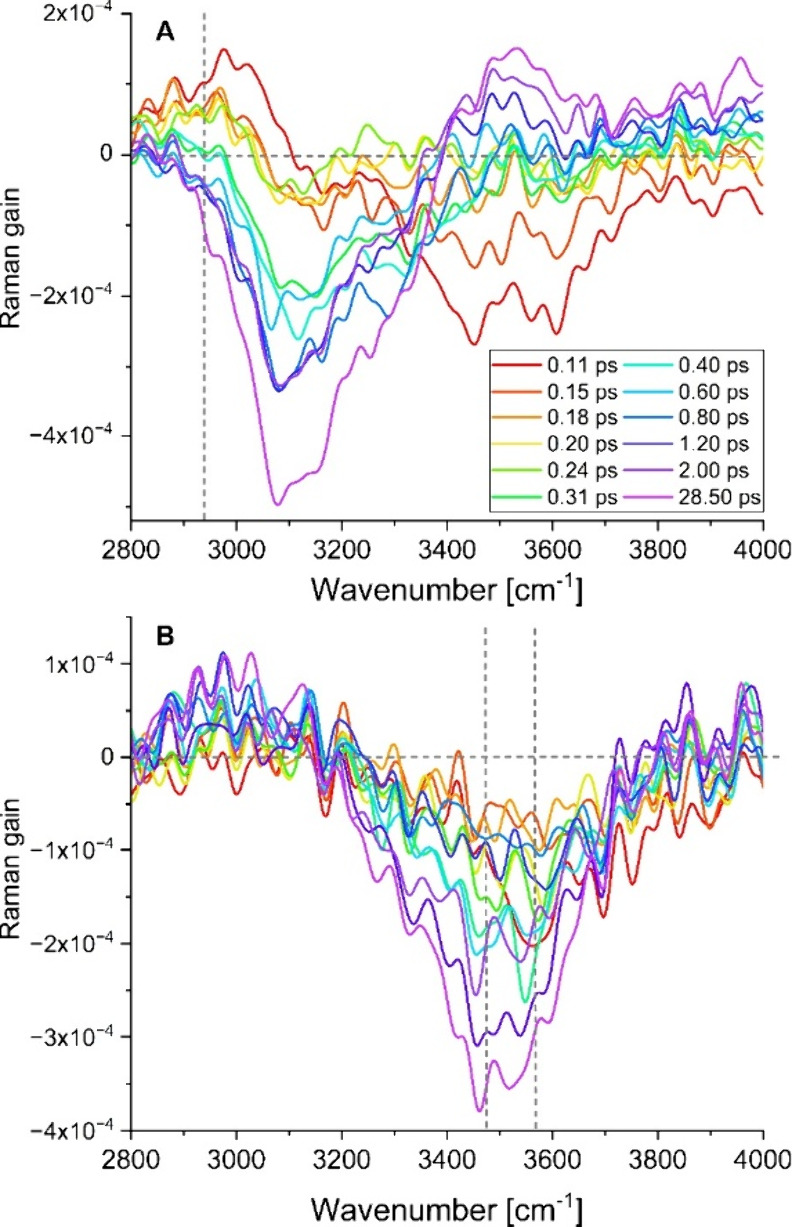
Transient fs-IR-SRS spectra after water excitation at 3950 cm^–1^ for (A) *pp* (polarized Raman) and (B) *sp* (depolarized Raman) polarization between Raman pump and Raman probe. The peak associated with OH stretch 1 → 2 vibrational transition in panel A and minima associated with 0 → 1 bleaching and thermalized signal in panel B are marked with dashed lines.

The rapid ingrowth of the strong HGS contribution to the transient spectra constitutes an obstacle to extracting the vibrational relaxation dynamics from it. Therefore, we performed the global fit to the spectra only in the range 2900–3200 cm^–1^, which is dominated by the decay of the 1→2 transition, and the HGS contribution is minor (Figure 3A). To extract OH stretching lifetimes *T*_1_, associated with the decay of excited state population, 1→0, through the rate constant *k*_1_ = 1/*T*_1_,^32,33^ a cascade kinetic model was used.^12,32^ The model describes the decay of the vibrationally excited OH stretch into a “dark” state (e.g., vibrational relaxation through bend overtone to bend fundamental mode) with the rate constant *k*_1__,_ which then decays to the low frequency modes with (k* = 1/T*) and results in the observation of the thermalized system characterized by HGS. From the free global fit of the *pp* polarization data shown in Figure 3A, we obtained ***T***_**1**_^***pp***^ = 200 ± 30 fs and T* = 550 ± 80 fs. This value of ***T***_**1**_^***pp***^ agrees with the results obtained by van der Post et al. for the red pump (200 fs, pump 3100 cm^–1^ – probe 2900 cm^–1^)^17^ and de Marco et al. (220 fs, pump 3400 cm^–1^ – probe in the range 3100–3400 cm^–1^),^19^ even though we pumped the OH stretch + libration combinational mode at 3950 cm^–1^. This indicates that the fast-relaxing, red-shifted components of the OH stretch in water were directly excited in our experiment. Furthermore, the thermalization kinetics determined in our experiments as 1/k*=T* = 550 ± 80 fs, matches the average value obtained by van der Post et al.,^17^ the earlier value reported by Lock and Bakker,^12^ and is only slightly faster than other reports (700–800 fs).^19,34^

**Figure 3:**
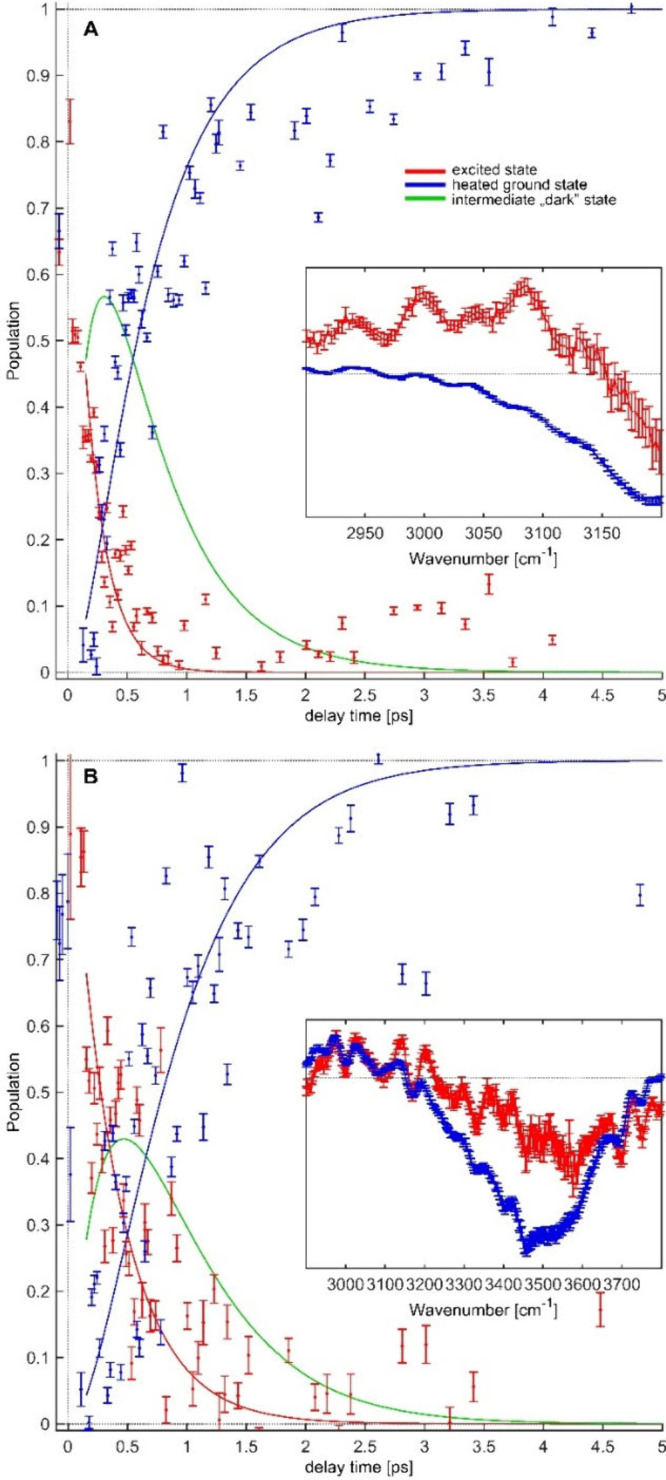
Population dynamics from the global fit of fs-IR-SRS studies of water for *pp* (A) and *sp* (B) polarization of the Raman probe. Points represent ES and HGS modeled spectra fitted to experimental data; solid lines denote kinetic fits. Insets: modeled associated decay spectra.

Figure 2B shows the transient *sp* (depolarized) Raman spectra of water for selected time delays. The bleaching of the Raman 0→1 transition dominates the spectra; however, some positive signals at around 2950 cm^–1^ (Raman 1→2 transition) are clearly visible. In the transient spectra, the bleach at early delay times (<0.4 ps) appears to be centered at around 3570 cm^–1^, and at longer delay times it shifts to below 3500 cm^–1^. By comparing the long-time-delay transient spectrum with the temperature-difference spectra for *sp* polarization (Figure S1d), it is clear that they match the difference spectra obtained after heating the water sample by 8 K, as observed for *pp* polarization. Interestingly, *sp* temperature difference spectra in this temperature range show only a negative signal below 3500 cm^–1^ (Raman cross-section decreasing with temperature) without any positive signals at longer wavelengths, as observed for higher temperatures. We thus conclude that the ingrowing spectral component centered below 3500 cm^–1^ is associated with water HGS for *sp* polarization.

The low signal-to-noise ratio of *sp* spectra makes an accurate estimation of vibrational lifetimes challenging. Because of the minuscule signals observed for the 1→2 transition around 2950 cm^–1^, we could not analyze the VER kinetics in that range, which would otherwise be useful in minimizing the contribution of thermal effects. Therefore, we applied a global fit to the *sp* Raman in the 2900–3800 cm^–1^ range using the same cascade kinetic model as for the *pp* Raman. To disentangle VER from the thermalization kinetics, we assumed T* to be the same as determined from *pp* experiments. We then fixed 1/k*=T* = 550 fs. The result of the global fit is shown in Figure 3B. Clearly, the ES decay for *sp* is significantly slower compared to the *pp* Raman. The vibrational relaxation time determined from the fit is ***T***_**1**_^***sp***^ = 400 fs vs ***T***_**1**_^***pp***^ = 200 ± 30 fs. It should be noted that the value of ***T***_**1**_^***sp***^ depends on the assumptions. The fit with the fewest number of constraints gives ***T***_**1**_^***sp***^=400 fs. If we additionally fix the shape of the HGS spectrum as an end transient spectrum, even slower kinetics of ***T***_**1**_^***sp***^ca. 900 fs can be obtained (see Figure S2). This is because we measured with a dense spacing of time delays at early time delays and a larger spacing at long time delays, so the HGS signal is statistically underrepresented in the obtained data. However, by varying fitting conditions, we always obtain ***T***_**1**_^***sp***^ values at least two times longer than that of ***T***_**1**_^***pp***^. Therefore, regardless of the assumptions, our results unambiguously demonstrate that OH stretch vibration relaxation in *sp* experiments is significantly slower, two times or more, than that of in *pp* experiments. According to numerous theoretical and experimental studies, the depolarized part of OH stretch is associated with uncoupled (out-of-phase) OH stretches,^22,23,27,35^ with some studies pointing at their more antisymmetric character.^24,25^

The high level of noise present in the IR pump *sp*-SRS probe transient spectra can be explained as follows. *sp* Raman spectra of the OH stretching band have an intensity of just around 20% of the *pp* signal at the OH stretch peak’s maximum. Considering that less than 2% of the water molecules in the irradiated volume are excited under the experimental conditions, this results in a very low signal-to-noise ratio for the transient spectra in this polarization configuration. Although we orient the liquid jet surface at the Brewster angle to the Raman probe to minimize its interference at the sample layer, the residual interference further affects the signal-to-noise ratio.

The very fast vibrational relaxation of the OH stretching mode is typically rationalized in terms of Fermi resonance with the first overtone of the bending mode 2*ν*_b_, which facilitates the intramolecular vibrational relaxation through the channel *ν*_s_ → 2*ν*_b_ → *ν*_b_. Moreover, Lock et al. demonstrated that vibrational relaxation through this channel slows down after water heating due to the thermal shift of *ν*_s_ to the blue, concurrent with the red shift of *ν*_b_, thereby minimizing resonance with 2*ν*_b_.^12^ Polarized Raman measures collective delocalized OH stretching modes, while depolarized Raman probes mainly uncoupled OH stretches. Therefore, the VER rate is likely associated with inter- and intramolecular coupling. What is more, the maximum of the depolarized OH stretching peak is strongly shifted to the blue (to *ca*. 3470 cm^–1^) compared to the polarized Raman band. Therefore, the OH stretches probed in *sp* experiments are detuned from the bend overtone and are thus off-Fermi resonance; they cannot relax directly through the same fast relaxation channel as lower-frequency OH stretches do.

To support the above conclusions derived from experimental results, we simulated the OH stretching lifetimes using a mixed quantum-classical approach in which classical molecular dynamics (MD) simulations were used to describe the low-frequency modes of liquid water, while the high-frequency modes, OH stretching and HOH bending overtones, were treated quantum-mechanically.^22,26,36^ The details of the method can be found in the SI. To simulate an experiment in which a broadband excitation is used, we calculated OH vibrational relaxation times by simultaneously exciting all OH stretching modes in the simulation box regardless of their hydrogen-bonding environments. The dynamics of the initial excitation were then monitored through the decay of the survival probability with time, from which *T*_1_ times were determined. Two types of simulations were performed. First, we ignored the Fermi coupling between OH stretching and HOH bending overtone and obtained *T*_1_ value of 263 fs. Including the Fermi coupling resulted in slightly faster vibrational relaxation of 251 fs. Both values, however, are in excellent agreement with the previously reported experimental value of 260 fs.^12^

Next, we investigated the excitation frequency dependence of the OH stretch vibrational relaxation. To this end, for a given excitation center frequency, all OH stretching modes whose vibrational frequencies were within 80 cm^–1^ from the center frequency were initially excited. The results are shown in Figure 4. Simulations were performed by including/excluding the coupling between the bend overtone modes and OH stretching modes. Only OH stretching modes were initially excited in both cases; however, due to the intramolecular Fermi coupling, in the former simulation, the relaxation is noticeably faster, being the fastest around 3200 cm^–1^. This is expected because the mixing between bend overtone and OH stretch is the strongest around 3250 cm^–1^.^26,37–39^ Simulations that include Fermi resonance yield vibrational lifetimes of 200 fs, in excellent agreement with our *pp*-polarized experiments. Without including Fermi resonance, the vibrational relaxation times are slightly slower at 220 fs for an excitation center frequency of 3200 cm^–1^. As is clear from Figure 4, the calculated vibrational relaxation times are essentially independent of the excitation frequency in the range 3100 – 3400 cm^–1^, changing from 200 fs at 3100 cm^–1^ to 204 fs at 3300 cm^–1^, and increasing only to 217 fs at 3400 cm^–1^. Such a weak dependence is in agreement with previous experiments for bulk water.^17^

**Figure 4:**
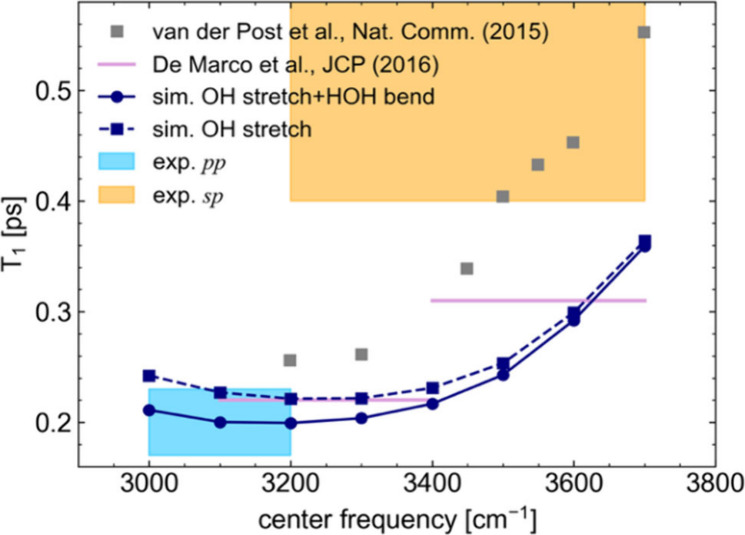
Experimental and simulated frequency-dependent OH-stretching lifetimes. Experimentally determined ***T***_**1**_^***pp***^ lifetime of 200 ± 30 fs is labeled “*pp*” and positioned at 3000–3200 cm^–1^. Experimental relaxation time ***T***_**1**_^***sp***^ ≥ 400 fs is labeled “*sp*” and is positioned at 3200–3700 cm^–1^ covering the range of the depolarized water OH stretching band. Simulated frequency-dependent vibrational energy relaxation times are reported for the calculations that include (solid dark blue) or neglect (dashed dark blue) OH-stretch-HOH bend overtone Fermi resonance. Previously reported experimental results from De Marco et al.^19^ and van der Post et al.^17^ are shown for comparison.

It should be noted that the kinetic model used to analyze experiments does not include energy transfer from the “dark” state back to the OH stretch. Our simulations allow for the energy transfer from the bending overtone to the OH stretch. A close agreement between simulations and experiments, however, suggests that the back transfer from bend overtone to OH stretch modes is much slower compared to the fast relaxation from the bend overtone to the bend fundamental.

The simulated lifetimes can be interpreted as follows. In 3100–3400 cm^–1^ frequency range OH stretch excitations are significantly delocalized. Indeed, the average molecular inverse participation ratio (IPR),^22,40^ which is the number of molecules over which the vibrational exciton is delocalized, in liquid water at 300 K is 18 at 3400 cm^–1^.^38^ IPR drops sharply to 5 at 3250 cm^–1^. OH stretching vibrations with frequencies near 3250 cm^–1^ are most strongly mixed with the bend overtone of the same molecule, facilitating very fast vibrational energy transfer between the modes. OH stretching vibrations with frequencies close but off-resonance with the bend overtone (*ca*. 3400 cm^–1^) are mixed with OH stretching vibrations in 18 other water molecules. Such delocalization allows the vibrational exciton to explore the large number of relaxation pathways very efficiently and, eventually, rapidly transfer to the bend overtone. This process, however, is impeded at higher frequencies. For 3600 cm^–1^, the molecular IPR is only 5, and the corresponding vibrations are far off-resonance with the bend overtone. Consequently, the VER times are longer and rapidly increase with increasing frequency: 300 fs at 3600 cm^–1^ and increasing to 360 fs at 3700 cm^–1^. Indeed, calculations show that at frequencies larger than 3500 cm^–1^ there is very little difference between the vibrational relaxation time with and without stretch–bend Fermi coupling.

To properly address the differences between the *T*_1_ values compared in Figure 4, we should carefully consider the experimental details of each of the cited works. The 2DIR experiment of De Marco et al. involved broadband excitation (pump bandwidth of 380 cm^–1^) centered at 3400 cm^–1^, and covering thus the entire OH stretching range, which was followed by an IR supercontinuum probe. Henceforth, the experiment provided an excellent time resolution, yet no spectral specificity in the excitation of OH stretches. Conceptually, the experiment is similar to our simulation with all OH stretches excited simultaneously, and determined in the experiment *T*_1_ of 220 fs for frequencies “below 3400 cm^–1^” and 310 fs “above 3400 cm^–1^” agrees well with our simulations. Their results serve as a qualitative indication of the frequency-dependence of *T*_1_, but should not be interpreted as a quantitative measure of that dependence.

On the other hand, van der Post et al.’s experiment provided excellent spectral specificity with pump pulses of 50 cm^–1^ bandwidth scanned through the entire OH stretching region, thus exciting selectively subensembles of OH stretches. They always probed the vibrational relaxation dynamics by analyzing the decay of excited state absorption in the range of 2800–3000 cm^–1^. Their experiment is conceptually comparable to our simulation, where the excitation center frequency is scanned through the range of 3000–3700 cm^–1^. These two data sets exhibit qualitatively similar frequency dependence, with van der Post et al.’s results consistently yielding longer *T*_1_ values than our simulations. The difference is most significant on the blue side of the OH stretch band (e.g., 550 fs vs 360 fs at 3700 cm^–1^). On the computational side, the discrepancy can be attributed to inaccuracies of spectroscopic maps or limitations of the classical water model used in MD simulations, which led to overestimating the population of OH stretching modes in the higher-frequency range. This can explain the faster relaxation times observed in simulations. Regarding van der Post’s experiment, we should note the 50 cm^–1^ pump pulse bandwidth, resulting in a pulse duration of around 300 fs for a Fourier-transform-limited pulse, which strongly affects the time resolution of their experiment.

Our IR pump – *pp*-polarized SRS probed studies, in which we determined ***T***_**1**_^***pp***^ = 200 ± 30 fs by probing similar spectral region as van der Post et al., generally agree with de Marco et al. for the probe below 3400 cm^–1^ (220 fs), van der Post et al. for the pump at 3100 cm^–1^ (200 fs) and our simulations in the range 3000–3400 cm^–1^ that accounted for the Fermi resonance (200–220 fs). The IR pump *sp*-polarized SRS probe studies estimated VER (***T***_**1**_^***sp***^> 400 fs) of the depolarized Raman component of the water OH stretching band, which is 1) centered at around 3470 cm^–1^ and 2) associated with the uncoupled fraction of the OH stretches. Hence, it perfectly agrees with the *T*_1_ determined by van der Post et al. for the pump at 3500 cm^–1^ (400 fs) and qualitatively agrees with the slowed VER observed by de Marco et al. and our simulations at the blue side of the OH stretching band. Nevertheless, by isolating the VER time of the blue-shifted decoupled OH stretches of water, our experiments provide information not accessible to other time-resolved methods that justifies the presence of frequency dependence in the OH stretch lifetimes. That is inter- and intramolecular decoupling and, simultaneously, a “spectral off-Fermi resonance” of the OH stretches located on the blue side of the band and the overtone of the bending mode.

It is essential to stress that the vibrational lifetimes heterogeneity of water presented in this work is only “spectral” – our results do not address the question of whether the structural heterogeneity of liquid water is long-living; the decoupled OH stretches are spatially distributed homogeneously in pure water. We should also emphasize that the conclusions of this work apply only to the bulk water. The experiments presented here do not also address the frequency-dependence of OH stretch lifetimes at the water/air interface discussed by van der Post et al.^17^ and Sung et al.^18^ However, we envision that an approach, similar to the one presented here, based on different polarization components of SFG spectroscopy can help determine the frequency-dependence of OH stretch lifetimes at interfaces.

## Experimental Section

### Fs-IR-SRS Spectroscopy

The fs-IR-SRS setup used in this study was a modification of the setup described elsewhere.^21^ Briefly, the source of the 200 fs pulses centered at 1030 nm, produced with the repetition rate 1 kHz was Yb:KGW Pharos laser (Light Conversion). The stimulated Raman gain signal was generated in the sample by the pair of pulses – narrowbandwidth (fwhm ≈ 5 cm^–1^) Raman pump (RPu) and broadband Raman probe (RPr). RPu around 3 ps long pulses, centered at 515 nm, were generated in the process of frequency mixing of two oppositely chirped copies of the 1030 nm laser pulse with the method described in ref 41. RPr was a femtosecond supercontinuum obtained by focusing less than 1 μJ of the 1030 nm beam in a Sapphire plate. The RPu and RPr were temporarily overlapped with a manual optical delay stage. Every second RPu pulse was blocked with a mechanical chopper (Thorlabs). The RPr spectra (RPu on/off) after the sample were detected using an Andor EMCCD Newton DU970P–FI camera attached to a Shamrock spectrograph SR 500i. The infrared pump pulses (IRp) with an energy of around 7 μJ and a duration of around 90 fs were generated by the home-built optical parametric amplifier described in ref 42. The beam waists (w_0_ = 1/e^2^) of the pumps at the focal points in the sample were approximately 20 μm. The IRp was delayed/advanced with respect to the pair of RPr and RPu using a precision optical delay line based on a motorized translation stage (Aerotech PRO165LM).

The ultrapure water sample was obtained from the Millipore Direct Q3 UV system. The isotopically diluted water was prepared by mixing H_2_O with D_2_O (Cambridge Isotope Laboratories Inc., 99.9% D atoms). The sample was delivered to the measurement spot of the setup by a homemade flow jet liquid sample cell. The speed of the gear pump was set to produce a liquid jet thickness of approximately 50 μm. The time-resolved measurements were performed at 20 °C. Stationary temperature-resolved studies were performed using the Harrick Temperature Control LiquidCell (TCE-S14–3) with a Watlow temperature controller.

### Data Analysis

Typically, we correct the RPu chirp-induced frequency dependence at time zero in broad-band fs-IR-SRS results. Such an operation, however, requires evenly spaced time delays, and we needed simultaneously high temporal resolution at early delays (<1 ps), acquisition of transient spectral data at long time delays (>100 ps) to properly determine thermalization kinetics, and multiple scans for improving the S/N ratio. Henceforth, we analyzed our data only in the narrow spectral range of 2900–3900 cm^–1^, without a chirp correction, since the chirp is approximately just 60 fs in that range, below the temporal resolution of the method (see Figure S3). The transient spectra were preprocessed using an FFT filter to remove the residual interference pattern of the RPu in a thin sample. The time-resolved data were analyzed using a global analysis based on the singular value decomposition method.

Error bars in Figure 3 are a result of error propagation from the standard error calculated for particular transient fs-IR-SRS spectra. Presented errors of *T*_1_ values are standard deviation values of at least 16 kinetic fits with various boundary conditions set for the global fit (start [0.1–0.3 ps] and end point [4–100 ps] of a fitted time window, spectral window ± 50 cm^–1^).

## Supplementary Material



## Notes

Temperature change during the experiment and temperature dependent stimulated Raman spectra of water, computational methods, Scheme S1, Figures S1–S3, and supplementary references (PDF)

## †

Ensemble^3^, Wólczyńska 133, 01-919 Warsaw, Poland

## Abbreviations

FSRS: femtosecond stimulated Raman scattering
VER: vibrational energy relaxation
HGS: heated ground state
GSB: ground state bleaching
ES: excited state
IPR: inverse participation ratio
fwhm: full-width at half of maximum

## References

[1] Vergauwe R. M. A., Thomas A., Nagarajan K., Shalabney A., George J., Chervy T., Seidel M., Devaux E., Torbeev V., Ebbesen T. W. (2019). Modification of Enzyme Activity by
Vibrational Strong Coupling of Water. Angew.
Chem., Int. Ed..

[2] Schatz G. C. (2000). Stretched
Water Is More Reactive. Science.

[3] Maillard J., Klehs K., Rumble C., Vauthey E., Heilemann M., Fürstenberg A. (2021). Universal quenching of common fluorescent probes by
water and alcohols. Chemical Science.

[4] Giubertoni G., Caporaletti F., Roeters S. J., Chatterley A. S., Weidner T., Laity P., Holland C., Woutersen S. (2022). In Situ Identification
of Secondary Structures in Unpurified Bombyx mori Silk Fibrils Using
Polarized Two-Dimensional Infrared Spectroscopy. Biomacromolecules.

[5] Ottosson N., Pastorczak M., van der Post S. T., Bakker H. J. (2014). Conformation of
the neurotransmitter γ-aminobutyric acid in liquid water. Phys. Chem. Chem. Phys..

[6] Yan C., Kramer P. L., Yuan R., Fayer M. D. (2018). Water Dynamics in
Polyacrylamide Hydrogels. J. Am. Chem. Soc..

[7] Fournier J. A., Carpenter W., De Marco L., Tokmakoff A. (2016). Interplay
of Ion-Water and Water-Water Interactions within the Hydration Shells
of Nitrate and Carbonate Directly Probed with 2D IR Spectroscopy. J. Am. Chem. Soc..

[8] Finney J. L. (2024). The structure
of water: A historical perspective. J. Chem.
Phys..

[9] Pakoulev A., Wang Z., Dlott D. D. (2003). Vibrational
relaxation and spectral
evolution following ultrafast OH stretch excitation of water. Chem. Phys. Lett..

[10] Wang Z., Pakoulev A., Pang Y., Dlott D. D. (2004). Vibrational Substructure
in the OH Stretching Transition of Water and HOD. J. Phys. Chem. A.

[11] Bakker H. J., Lock A. J., Madsen D. (2004). Comment on
‘Vibrational relaxation
and spectral evolution following ultrafast OH stretch excitation of
water’ by A. Pakoulev, Z. Wang, D.D. Dlott. Chem. Phys. Lett..

[12] Lock A. J., Bakker H. J. (2002). Temperature dependence
of vibrational relaxation in
liquid H_2_O. J. Chem. Phys..

[13] Lindner J., Vöhringer P., Pshenichnikov M. S., Cringus D., Wiersma D. A., Mostovoy M. (2006). Vibrational
relaxation of pure liquid water. Chem. Phys.
Lett..

[14] Ramasesha K., De Marco L., Mandal A., Tokmakoff A. (2013). Water vibrations
have strongly mixed intra- and intermolecular character. Nat. Chem..

[15] Cowan M. L., Bruner B. D., Huse N., Dwyer J. R., Chugh B., Nibbering E. T. J., Elsaesser T., Miller R. J. D. (2005). Ultrafast memory
loss and energy redistribution in the hydrogen bond network of liquid
H_2_O. Nature.

[16] Kraemer D., Cowan M. L., Paarmann A., Huse N., Nibbering E. T. J., Elsaesser T., Miller R. J. D. (2008). Temperature dependence of the two-dimensional
infrared spectrum of liquid H_2_O. Proc. Natl. Acad. Sci. U. S. A..

[17] van
der Post S. T., Hsieh C.-S., Okuno M., Nagata Y., Bakker H. J., Bonn M., Hunger J. (2015). Strong frequency dependence
of vibrational relaxation in bulk and surface water reveals sub-picosecond
structural heterogeneity. Nat. Commun..

[18] Sung W., Inoue K.-i., Nihonyanagi S., Tahara T. (2024). Unified picture of
vibrational relaxation of OH stretch at the air/water interface. Nat. Commun..

[19] De
Marco L., Fournier J. A., Thämer M., Carpenter W., Tokmakoff A. (2016). Anharmonic exciton dynamics and energy
dissipation in liquid water from two-dimensional infrared spectroscopy. J. Chem. Phys..

[20] Pastorczak M., Nejbauer M., Radzewicz C. (2019). Femtosecond
infrared pump-stimulated
Raman probe spectroscopy: the first application of the method to studies
of vibrational relaxation pathways in the liquid HDO/D_2_O system. Phys. Chem. Chem. Phys..

[21] Pastorczak M., Nejbauer M., Kardas T., Radzewicz C. (2019). Femtosecond
infrared pump - stimulated Raman probe spectroscopy: the method and
its first application to study vibrational relaxation pathway in liquid
water. EPJ. Web Conf..

[22] Auer B. M., Skinner J. L. (2008). IR and Raman spectra of liquid water:
Theory and interpretation. J. Chem. Phys..

[23] Bakker H. J., Skinner J. L. (2010). Vibrational Spectroscopy
as a Probe of Structure and
Dynamics in Liquid Water. Chem. Rev..

[24] Walrafen G.
E. (2006). Contribution
of the asymmetric stretch, ν_3_B_1_, to the
fundamental Raman spectrum of water. J. Chem.
Phys..

[25] Murphy W. F., Bernstein H. J. (1972). Raman spectra
and an assignment of the vibrational
stretching region of water. J. Phys. Chem..

[26] Kananenka A. A., Skinner J. L. (2018). Fermi resonance in OH-stretch vibrational spectroscopy
of liquid water and the water hexamer. J. Chem.
Phys..

[27] Ishihara S., Takayama T., Sakaguchi M., Otosu T., Yagasaki T., Suzuki Y., Yamaguchi S. (2022). Raman spectroscopy
of isotopically
pure and diluted high- and low-density amorphous ices. J. Raman Spectrosc..

[28] De
Marco L., Carpenter W., Liu H., Biswas R., Bowman J. M., Tokmakoff A. (2016). Differences in the Vibrational Dynamics
of H_2_O and D_2_O: Observation of Symmetric and
Antisymmetric Stretching Vibrations in Heavy Water. J. Phys. Chem. Lett..

[29] Collins J. R. (1939). A New Infra-red
Absorption Band of Liquid Water at 2.52μ. Phys. Rev..

[30] Pastorczak M., Duk K., Shahab S., Kananenka A. A. (2023). Combinational Vibration Modes in
H_2_O/HDO/D_2_O Mixtures Detected Thanks to the
Superior Sensitivity of Femtosecond Stimulated Raman Scattering. J. Phys. Chem. B.

[31] Lock A. J., Woutersen S., Bakker H. J. (2001). Ultrafast Energy Equilibration in
Hydrogen-Bonded Liquids. J. Phys. Chem. A.

[32] Nienhuys H.-K., Woutersen S., van Santen R. A., Bakker H. J. (1999). Mechanism for vibrational
relaxation in water investigated by femtosecond infrared spectroscopy. J. Chem. Phys..

[33] Laubereau A., von der Linde D., Kaiser W. (1972). Direct Measurement of the Vibrational
Lifetimes of Molecules in Liquids. Phys. Rev.
Lett..

[34] Carpenter W. B., Fournier J. A., Biswas R., Voth G. A., Tokmakoff A. (2017). Delocalization
and stretch-bend mixing of the HOH bend in liquid water. J. Chem. Phys..

[35] Yang M., Skinner J. L. (2010). Signatures of coherent
vibrational energy transfer
in IR and Raman line shapes for liquid water. Phys. Chem. Chem. Phys..

[36] Auer B. M., Skinner J. L. (2007). Dynamical effects
in line shapes for coupled chromophores:
Time-averaging approximation. J. Chem. Phys..

[37] Hunter K. M., Shakib F. A., Paesani F. (2018). Disentangling Coupling Effects in
the Infrared Spectra of Liquid Water. J. Phys.
Chem. B.

[38] Kananenka A. A., Hestand N. J., Skinner J. L. (2019). OH-Stretch Raman
Multivariate Curve
Resolution Spectroscopy of HOD/H_2_O Mixtures. J. Phys. Chem. B.

[39] Lüttschwager N.
O. B. (2024). The
strength of the OH-bend/OH-stretch Fermi resonance in small water
clusters. Phys. Chem. Chem. Phys..

[40] Thouless D.
J. (1974). Electrons
in disordered systems and the theory of localization. Phys. Rep..

[41] Nejbauer M., Radzewicz C. (2012). Efficient spectral shift and compression
of femtosecond
pulses by parametric amplification of chirped light. Opt. Express.

[42] Nejbauer M., Kardaś T. M., Pastorczak M., Radzewicz C. (2023). Broadly tunable
femtosecond OPA pumped by 1030 nm Yb: KGW laser with a range of non-linear
crystals tested. Infrared Physics & Technology.

