# Supplementary material for: The Origin of the Frequency-Dependence of OH Stretch Vibrational Lifetimes in Liquid Water
Source: J Phys Chem Lett. 2026 Jul 27;17(31):8790–7. doi: 10.1021/acs.jpclett.6c00970 (PMC13455094; doi:10.1021/acs.jpclett.6c00970)
Supplement: Supplementary file 1 [file jz6c00970_si_001.pdf]

Supporting Information for

The origin of the frequency-dependence of OH stretch vibrational lifetimes in liquid water

Marcin Pastorczak[1]*, Czesław Radzewicz[1,2], Aritri Biswas[3], and Alexei A. Kananenka[3†]

[1] *Institute of Physical Chemistry, Polish Academy of Sciences, Kasprzaka 44/52, 01-224 Warsaw, Poland*

[2] *Institute of Experimental Physics, Faculty of Physics, University of Warsaw, Pasteura 5, 02-093 Warsaw, Poland*

[3] *Department of Physics and Astronomy, University of Delaware, Newark, Delaware 19716, United States*

*Marcin Pastorczak
Email: mpastorczak@ichf.edu.pl

†Alexei A. Kananenka
Email: akanane@udel.edu

This PDF file includes:

1. Supporting text - Temperature change during the experiment and temperature dependent stimulated Raman spectra of water.
2. Supporting text – Computational methods
3. Scheme S1
4. Figures S1 to S3
5. Supplementary References

1. Temperature change during the experiment and temperature dependent stimulated Raman spectra of water.

The magnitude of the temperature increase associated with the IR pump energy in the fs-IR-SRS experiment (around 8 K) was calculated with use of the following formula [1]:

$$\Delta T = \frac{k}{\rho C_p}\left(\frac{I}{A}\right)^{-kz}$$

,where $k = 100\ cm^{-1}$ is the absorption coefficient of water at 2530 nm, the beam of energy per pulse I=0.000007 *J* has a waist w=1/e²= 0.002 *cm*, and the sample thickness z = 0.005 *cm* is, what gives the area of sample irradiated by the infrared pump pulse $A = 1.257 \cdot 10^{-5}\ cm^2$ $C_p \approx 4.18\ [J/g \cdot K]$ is the heat capacity and $r \approx 1 \frac{g}{cm^3}$ density of water at room temperature.

2. Computational methods

Molecular dynamics simulations

Classical molecular dynamics (MD) simulations were performed using three-body water model E3B2.[2] It has been shown that, together with the mixed quantum-classical approach described below, E3B2 water model provides IR and Raman spectra of liquid water in a very good agreement with experiment.[3] MD simulations were performed in the NVT ensemble with 500 H_2O E3B2 molecules using GROMACS package version 4.5.5[4] modified to implement an E3B2 model. The simulation box size was scaled to reproduce the experimental density of liquid H_2O at 298 K. Three-dimensional periodic boundary conditions were applied. Electrostatic interactions were calculated using the particle-mesh Ewald summation.[5] The Lennard-Jones interactions were truncated at 0.95 nm. The Nose-Hoover algorithm[6, 7] with a 2 ps coupling parameter was used to maintain the system at the constant temperature 298 K. The classical equations of motion were propagated with a 1 fs time step using the SETTLE algorithm.[8] After an equilibration run of 1.0 ns, the production run of 2.0 ns was performed. During the production run, the atomic coordinates were saved every 10 fs and used for calculating OH-stretch vibrational relaxation times as described below.

OH-stretch vibrational relaxation dynamics were calculated using mixed quantum-classical approach which has been described in detail elsewhere.[3, 9, 10] Briefly, the main idea of the approach is to partition all degrees of freedom in the system into the subsystem of interests, usually a collection of fast degrees of freedom and the environment (bath) which is composed of slow degrees of freedom. In this work the subsystem of interests consists of OH stretch and HOH bend overtone modes which are treated quantum-mechanically as described below. The bath includes low-frequency degree of freedom are associated with translations, rotations, and low-frequency vibrations and are treated classically using MD simulations as described above.

In the quantum-mechanical treatment, the system of N vibrational chromophores (OH stretches and HOH bend overtones in this work) is described by the one-exciton model. In the local mode basis, the vibrational exciton Hamiltonian is given by

$$H(t) = H_0|0\rangle\langle 0| + \sum_{i=1}^{N} \hbar\omega_i(t)|i\rangle\langle i| + \sum_{i,j=1;\, i\neq j}^{N} \hbar\omega_{ij}(t)|i\rangle\langle j| \qquad (1)$$

This Hamiltonian describes a system of vibrational chromophores with the total ground state $|0\rangle$ where all chromophores are in their ground states. The second term in Eq. (1) describes excitations of each of N chromophores, where $|i\rangle$ is the state of the system in which only ith chromophore, which can be OH stretch or HOH bend overtone, is excited. For OH stretch mode the transition frequency $\omega_i(t)$ corresponds to the 0-1 excitation frequency while for HOH bend overtone it is the 0-2 excitation frequency of the HOH bending mode. The third term in Eq. (1) contains the couplings $\omega_{ij}(t)$, where i and j denote OH stretch or HOH bend overtone modes. Note that OH stretch couplings can be either intramolecular or intermolecular.

The time-dependence of the transition frequencies $\omega_i(t)$ and couplings $\omega_{ij}(t)$ originates from fluctuations of the classical bath and $\omega_{ij}(t)$. The dependence of the transition frequencies on the bath modes is modeled using previously developed spectroscopic maps. The OH stretch frequency is modeled as a polynomial function of the electric field taken from Ref.[11]: $\omega_{OH,i}(E_i) = 3760.2 - 3541.7E_i - 152\,677E_i^2$, where E_i is the projection of the electric field onto the OH bond of the ith chromophore.[9, 11-14] The electric field is calculated for each configuration of water molecules at each time step of MD simulation from the point charges utilized in E3B2 water model. The intramolecular OH stretch couplings are described by the coupling map from Ref.[11]

$$\omega_{ij}(E_i, E_j) = \left(-1361 + 27165(E_i + E_j)x_i x_j - 1.887 p_i p_j\right) \qquad (2)$$

where E_i is the projection of the electric field on the OH bond of the ith chromophore, $x_i = 0.19285 - 1.7261 \times 10^{-5}\omega_i$ and $p_i = 1.6466 + 5.7692 \times 10^{-4}\omega_i$ are the 0-1 position and momentum matrix elements of ith OH stretching mode.[11] Bend overtone transition frequencies were described by the frequency map from Ref.[3]: $\omega_{HOH} = 3132.78 + 6086.31E$. Here E is the sum of the components of electric fields perpendicular to the two OH bonds and pointing toward the HOH bisector, calculated at the positions of the hydrogen atoms.[15]

Intermolecular coupling between OH stretching modes i and j is assumed to be described by the transition dipole interaction[11, 16] $\hbar\omega_{ij} = k_{ij}x_i x_j$, where

$$k_{ij} = \frac{\mu_i'\mu_j'\{u_i \cdot u_j - 3[(u_i \cdot n_{ij})(u_j \cdot n_{ij})]\}}{r_{ij}^3} \tag{3}$$

where μ_i' is the magnitude of the dipole derivative, u_i is the unit vector along the direction of the transition dipole, n_{ij} is the unit vector along the line connecting the location of OH stretch transition dipoles i and j and r_{ij} is the distance between these locations. The transition dipole of an OH stretching oscillator is located along the OH bond 0.67 Å away from the oxygen atom.[17] The dipole derivative map used in this work is taken from Ref.[11]: $\mu_i'(E_i) = 0.1646 + 11.39E_i + 63.41E_i^2$, where E_i is the projection of the electric field onto the OH bond of the ith chromophore.

The intermolecular coupling between the fundamental HOH bending modes is known to be proportional to the product of the 0-1 transition matrix elements.[15] Because the 0-2 transition matrix element is smaller than 0-1, the intermolecular coupling between HOH bending overtone modes is neglected. Following our previous work,[3] the Fermi resonance coupling between OH stretch and HOH bending overtone was set to 25 cm^{-1}.

The OH stretch vibrational lifetime was determined as follows.[18, 19] Suppose that the initial state of the system is the excitation at ith site, $|i\rangle$, which can be either a subset of OH stretches in a narrow-band excitation or all OH stretches in a broad-band excitation. The state vector, which encodes the state of the system at time t later, given that the state $|i\rangle$ was initially excited, is $U(t)|i\rangle$. $U(t)$ is a quantum-mechanical propagator that obeys the time-dependent Schrödinger equation

$$\frac{d}{dt}U(t) = -\frac{i}{\hbar}H(t)U(t) \tag{4}$$

where $H(t)$ is the excitonic Hamiltonian, Eq. (1). The amplitude of finding the excitation on site j at time t, for a particular trajectory, is given by $\langle j|U(t)|i\rangle \equiv U_{ji}(t)$. Note that because OH stretching modes are coupled inter- and intramolecularly, as well as to the HOH bending modes, the initial excitation of OH stretching modes will populate bending overtone modes. Therefore, j in $U_{ji}(t)$ denotes both OH stretching and HOH bend overtone modes. The average probability of finding the excitation on site j at time t, given that it was initially at site i, is $P_{ji}(t) = \left\langle \left|U_{ji}(t)\right|^2 \right\rangle$, where the brackets denote a statistical average over an ensemble of trajectories. The average survival probability can now be written in terms of $P_{ii}(t)$ as

$$S(t) = \frac{1}{N}\sum_{i=1}^{N} P_{ii}(t) \tag{5}$$

where N is the number of OH stretch chromophores in the system. $S(t)$ decays over time as the initial excitation is spread over other modes due to inter- and intramolecular couplings. The average OH stretching lifetimes were computed by initially exciting all $2N$ (for N water molecules) OH stretch modes. Experimentally this corresponds to a broad-band excitation. Frequency-dependent OH stretch relaxation times were determined by initially exciting only those OH stretching modes whose frequency were within an experimental narrow-band excitation range of $\omega_c \pm 80$ (cm^{-1}), for various

center frequencies ω_c as reported in the main text. The survival probability was calculated by monitoring $U_{ii}(t)$ where index i corresponds to OH stretching modes (bending overtones are omitted). All reported OH stretch lifetimes were calculated by integrating the survival probability $T_1 = \int_0^\infty dt S(t)$ averaged over 100 trajectories each 1.5 ps long sampled from MD simulations. The impact of OH stretch-HOH bend Fermi resonance on OH stretching lifetimes was investigated by including the Fermi coupling between the fundamental OH and overtone of HOH bend modes as described above. All calculations were performed using MultiSpec package which is freely available at: https://github.com/kananenka-group/MultiSpec.

We note that the rate of the vibrational energy transfer can also be estimated using Forster theory, as was done in Ref.[18, 19] but as was also shown in that work, the mixed quantum-classical approach used here provides better agreement with experiment (anisotropy decay).

3. Scheme

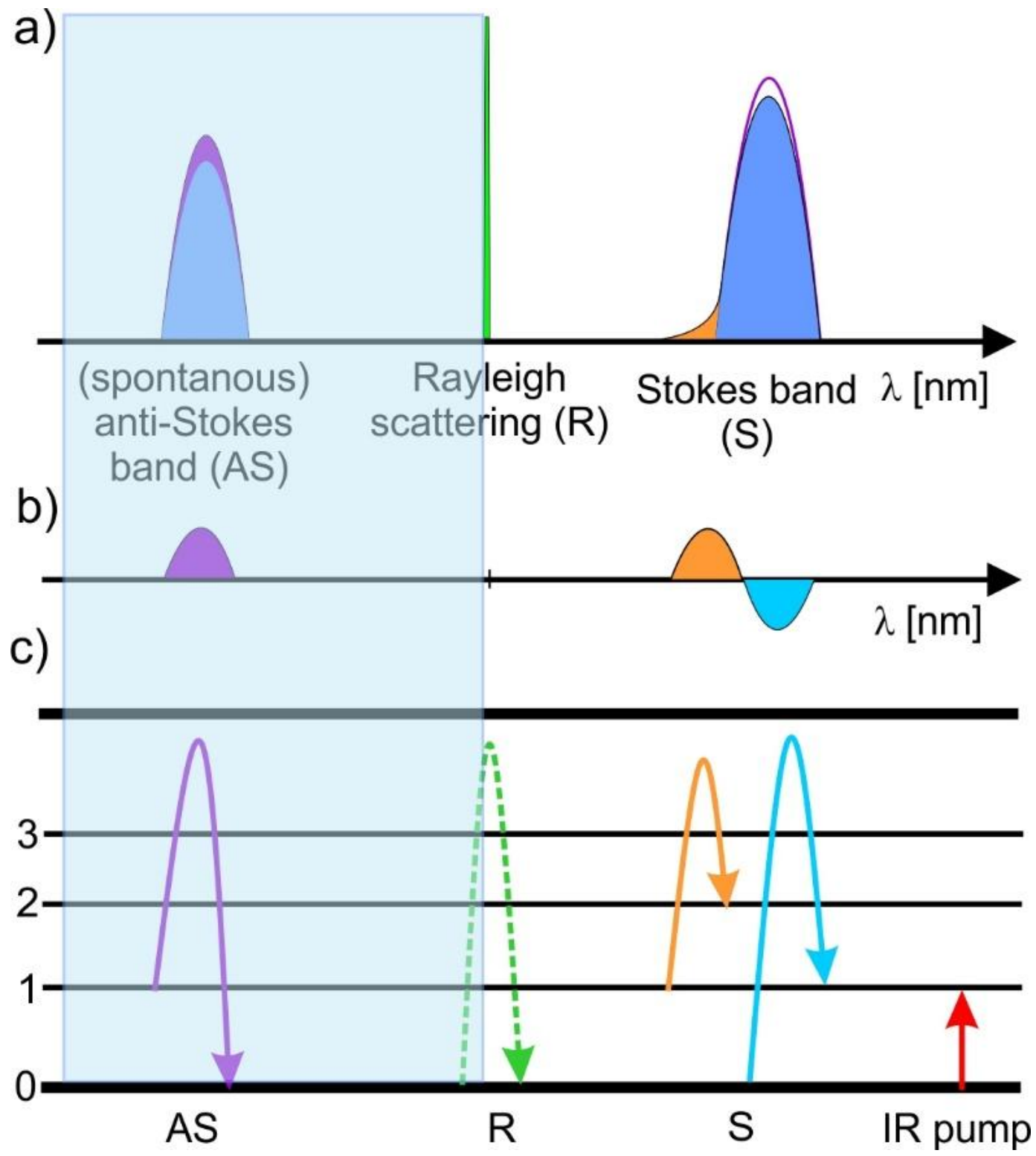

Scheme 1 - Schematic representation of the Raman processes after excitation of vibrational transition 0→ 1 in a molecule with an IR pulse d), stationary Raman spectrum (a) changes in the Raman spectrum (b) and in transient Raman spectrum (c) induced by a depopulation of the vibrational ground state and increase of population of the first excited state. R denoted Rayleigh scattering, and S denote Stokes Raman scattering. Note that shown anti-Stokes part (blue shaded) refers only to spontaneous Raman; stimulated Raman scattering observed at the blue ("anti-Stokes") side of the Raman pump involves also vibrational 0→ 1 transition with the same scheme as shown for the red ("Stokes") side.

4. Figures

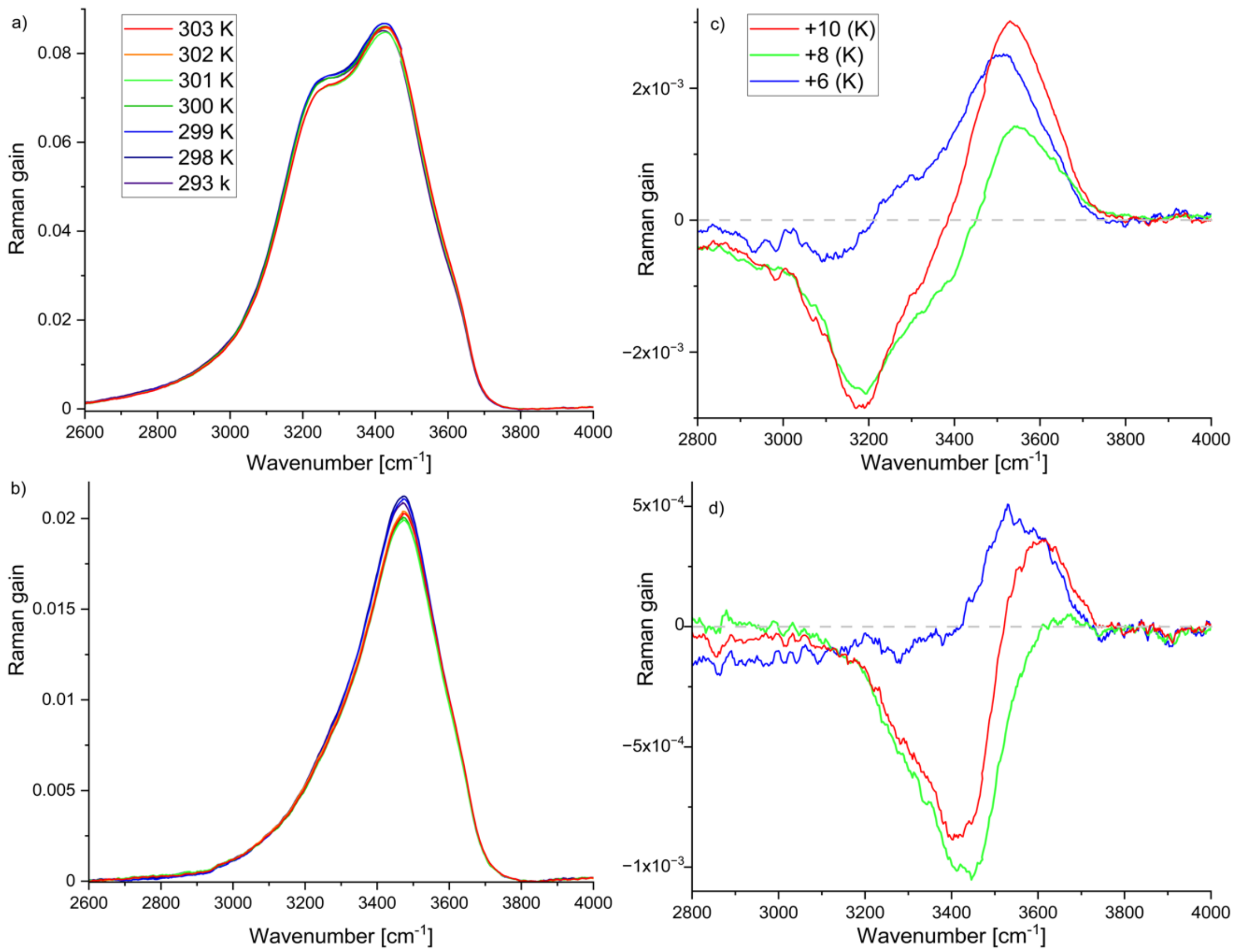

Fig. S1. temperature dependent stationary stimulated Raman spectra of water for *pp* (a) and *sp* polarizations (b) and difference spectra calculated as a difference between spectrum at given temperature minus spectrum at room temperature; c) for *pp* and d) for *sp* polarization. In the legend for panel c) and d) the increase in temperature from room temperature is shown.

Please note: the difference spectra (panels c) and d)) present only qualitative similarity of difference spectra profiles obtained for +8 K to the modeled HGS spectrum from time-resolved experiments. They should not be interpreted quantitatively due to the low precision (1K) of the temperature controller of the heating cell and the presence of baseline originating from temperature cell windows, the subtraction of which is problematic.

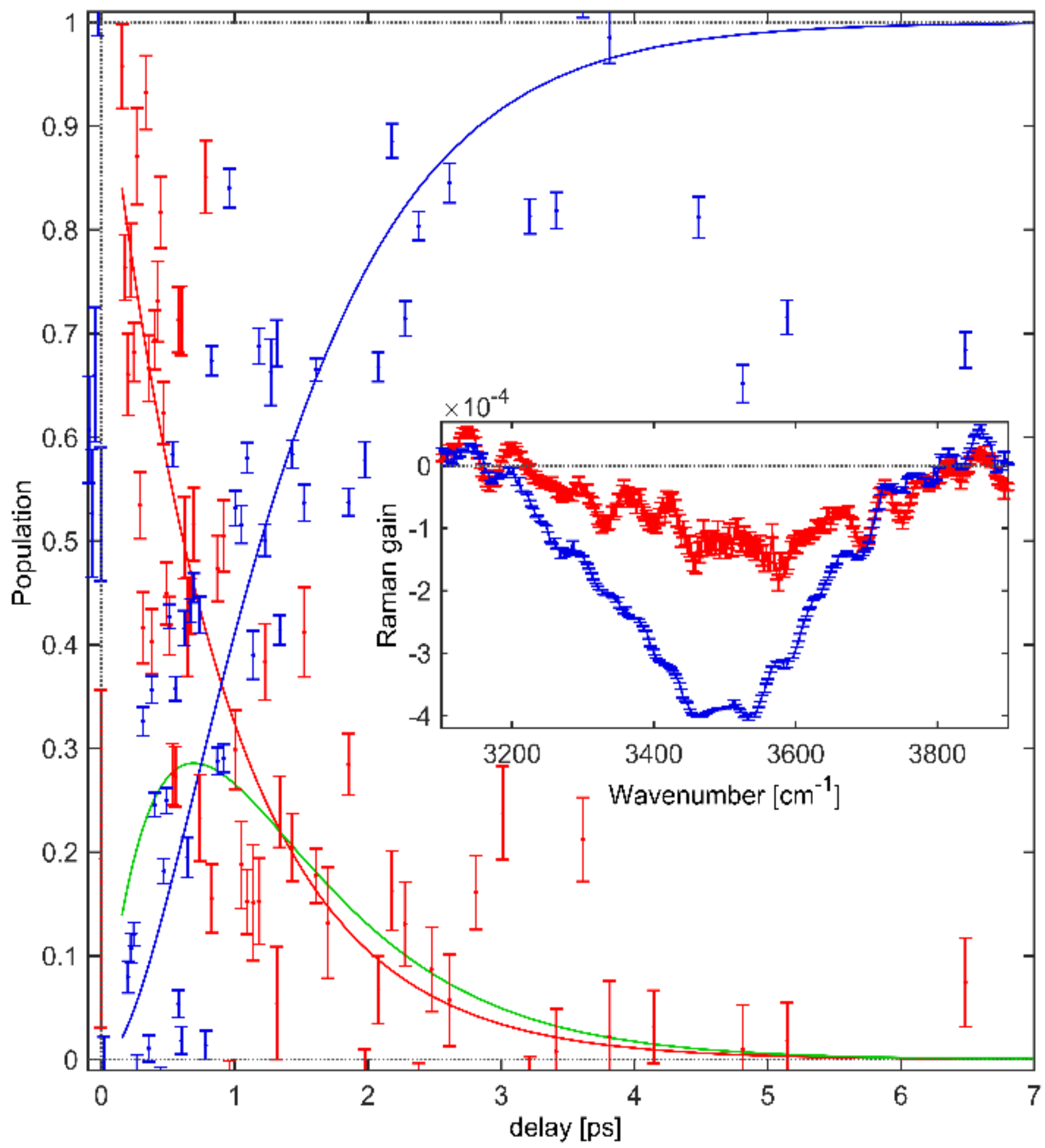

Fig. S2. Population dynamics from the global fit of fs-IR-SRS studies of water for *sp* polarization of the Raman probe. Fit with fixed both thermalization kinetics *1/k**=550 fs and HGS spectrum as a spectrum between 40 ps and 130 ps, resulting in the fitted $\boldsymbol{T_1^{sp}}$= 900 ± 50 fs. Insets: modelled decay associated spectra.

We verified also the result of the above presented fit with T* value fixed within its errorbar (± 80 fs). In such a case we obtained $\boldsymbol{T_1^{sp}}$ = 1.06 ps for T* =470 fs and $\boldsymbol{T_1^{sp}}$ = 760 fs for T*=630 fs, and in the case of the fit without fixing the HGS spectrum (shown in Figure 3 in the manuscript), the result is as follows:
for T* = 470 fs, $\boldsymbol{T_1^{sp}}$ = 480 fs, and for T*= 630 fs, $\boldsymbol{T_1^{sp}}$=320 fs.

These additional fits still support our hypothesis on $\boldsymbol{T_1^{sp}}$ being at least twice as long as $\boldsymbol{T_1^{pp}}$ (200 ± 30 fs).

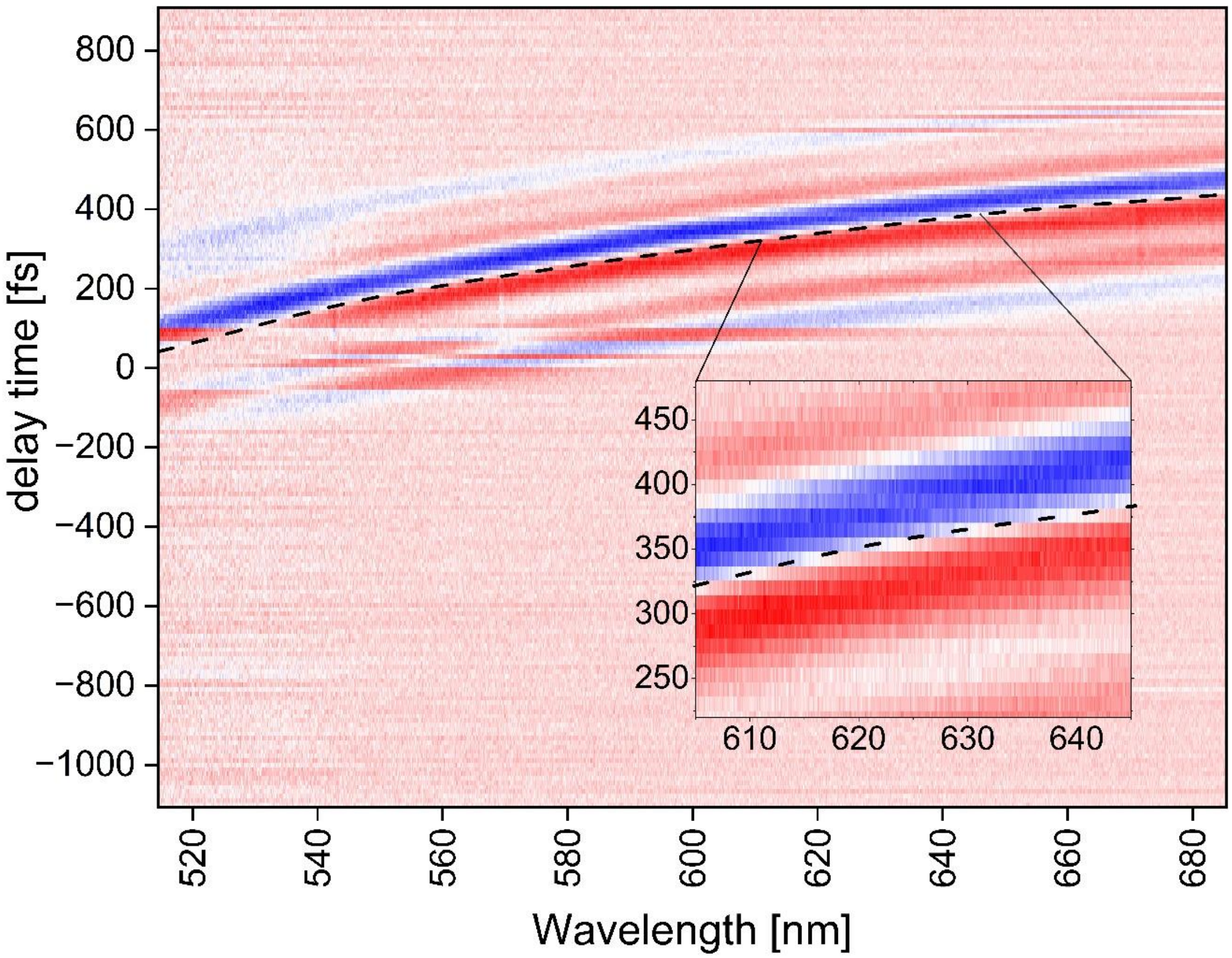

Fig. S3. The cross-phase modulation artefact (XPM) between the white light (RPr) and the IR pulse (IRp) in the range of the measured stimulated Raman spectra. The inset shows the zoom-in on the range 605-645 nm which corresponds to the range 2900-3900 cm^{-1} of the fs-IR-SRS spectra analyzed in this work. The black dashed line marks the zero-crossing of the XPM, which corresponds to the time "zero" of the IR pump SRS probe experiment [20]. Hot and cold colors stand for positive and negative XPM values, respectively.

SI References

(1) Kubelka, J. Time-resolved methods in biophysics. 9. Laser temperature-jump methods for investigating biomolecular dynamics. *Photochemical & Photobiological Sciences* **2009**, *8* (4), 499-512, DOI: 10.1039/b819929a.

(2) Tainter, C. J.; Pieniazek, P. A.; Lin, Y.-S.; Skinner, J. L. Robust three-body water simulation model. *The Journal of Chemical Physics* **2011**, *134* (18). DOI: 10.1063/1.3587053

(3) Kananenka, A. A.; Skinner, J. L. Fermi resonance in OH-stretch vibrational spectroscopy of liquid water and the water hexamer. *The Journal of Chemical Physics* **2018**, *148* (24), 244107. DOI: 10.1063/1.5037113.

(4) Hess, B.; Kutzner, C.; van der Spoel, D.; Lindahl, E. GROMACS 4: Algorithms for Highly Efficient, Load-Balanced, and Scalable Molecular Simulation. *Journal of Chemical Theory and Computation* **2008**, *4* (3), 435-447. DOI: 10.1021/ct700301q.

(5) Darden, T.; York, D.; Pedersen, L. Particle mesh Ewald: An N·log(N) method for Ewald sums in large systems. *The Journal of Chemical Physics* **1993**, *98* (12), 10089-10092. DOI: 10.1063/1.464397

(6) Nosé, S. A unified formulation of the constant temperature molecular dynamics methods. *The Journal of Chemical Physics* **1984**, *81* (1), 511-519. DOI: 10.1063/1.447334

(7) Hoover, W. G. Canonical dynamics: Equilibrium phase-space distributions. *Physical Review A* **1985**, *31* (3), 1695-1697. DOI: 10.1103/PhysRevA.31.1695.

(8) Miyamoto, S.; Kollman, P. A. Settle: An analytical version of the SHAKE and RATTLE algorithm for rigid water models. *Journal of Computational Chemistry* **1992**, *13* (8), 952-962. DOI: https://doi.org/10.1002/jcc.540130805.

(9) Auer, B. M.; Skinner, J. L. IR and Raman spectra of liquid water: Theory and interpretation. *The Journal of Chemical Physics* **2008**, *128* (22), 224511. DOI: 10.1063/1.2925258.

(10) Auer, B. M.; Skinner, J. L. Dynamical effects in line shapes for coupled chromophores: Time-averaging approximation. *The Journal of Chemical Physics* **2007**, *127* (10). DOI: 10.1063/1.2766943

(11) Gruenbaum, S. M.; Tainter, C. J.; Shi, L.; Ni, Y.; Skinner, J. L. Robustness of Frequency, Transition Dipole, and Coupling Maps for Water Vibrational Spectroscopy. *Journal of Chemical Theory and Computation* **2013**, *9* (7), 3109-3117. DOI: 10.1021/ct400292q.

(12) Li, F.; Skinner, J. L. Infrared and Raman line shapes for ice Ih. I. Dilute HOD in H2O and D2O. *The Journal of Chemical Physics* **2010**, *132* (20). DOI: 10.1063/1.3430518

(13) Auer, B.; Kumar, R.; Schmidt, J. R.; Skinner, J. L. Hydrogen bonding and Raman, IR, and 2D-IR spectroscopy of dilute HOD in liquid D_2O. *Proceedings of the National Academy of Sciences* **2007**, *104* (36), 14215-14220. DOI: doi:10.1073/pnas.0701482104.

(14) Corcelli, S. A.; Lawrence, C. P.; Skinner, J. L. Combined electronic structure/molecular dynamics approach for ultrafast infrared spectroscopy of dilute HOD in liquid H_2O and D_2O. *The Journal of Chemical Physics* **2004**, *120* (17), 8107-8117. DOI: 10.1063/1.1683072

(15) Ni, Y.; Skinner, J. L. IR and SFG vibrational spectroscopy of the water bend in the bulk liquid and at the liquid-vapor interface, respectively. *The Journal of Chemical Physics* **2015**, *143* (1). DOI: 10.1063/1.4923462

(16) Li, F.; Skinner, J. L. Infrared and Raman line shapes for ice Ih. II. H_2O and D_2O. *The Journal of Chemical Physics* **2010**, *133* (24). DOI: 10.1063/1.3516460

(17) Pieniazek, P. A.; Tainter, C. J.; Skinner, J. L. Surface of Liquid Water: Three-Body Interactions and Vibrational Sum-Frequency Spectroscopy. *Journal of the American Chemical Society* **2011**, *133* (27), 10360-10363. DOI: 10.1021/ja2026695.

(18) Shi, L.; Li, F.; Skinner, J. L. Resonant vibrational energy transfer in ice Ih. *The Journal of Chemical Physics* **2014**, *140* (24). DOI: 10.1063/1.4883913

(19) Yang, M.; Li, F.; Skinner, J. L. Vibrational energy transfer and anisotropy decay in liquid water: Is the Förster model valid? *The Journal of Chemical Physics* **2011**, *135* (16). DOI: 10.1063/1.3655894

(20) Pastorczak, M.; Nejbauer, M.; Radzewicz, C. Femtosecond infrared pump–stimulated Raman probe spectroscopy: the first application of the method to studies of vibrational relaxation pathways

in the liquid HDO/D_2O system. *Physical Chemistry Chemical Physics* **2019**, *21* (31), 16895-16904,. DOI: 10.1039/c9cp00855a.
